# The prevalence of *Leptospira* among invasive small mammals on Puerto Rican cattle farms

**DOI:** 10.1371/journal.pntd.0007236

**Published:** 2019-05-20

**Authors:** Kathryn M. Benavidez, Trina Guerra, Madison Torres, David Rodriguez, Joseph A. Veech, Dittmar Hahn, Robert J. Miller, Fred V. Soltero, Alejandro E. Pérez Ramírez, Adalberto Perez de León, Iván Castro-Arellano

**Affiliations:** 1 Department of Biology, Texas State University, San Marcos, Texas, United States of America; 2 Cattle Fever Tick Research Laboratory, United States Department of Agriculture–Agricultural Research Service, MAB 6419, Edinburg, Texas, United States of America; 3 Animal and Plant Health Inspection Service PR and USVI SPRS District 2, United States Department of Agriculture, Hato Rey, Puerto Rico; 4 Agrological Laboratory Analysis & Registration of Agricultural Materials PR Dept. of Agriculture 7 Carr. 693 Dorado, PR; 5 Knippling-Bushland U.S. Livestock Insects Research Laboratory, United States Department of Agriculture–Agricultural Research Service, Kerrville, Texas, United States of America; 6 Veterinary Pest Genomics Center, United States Department of Agriculture–Agricultural Research Service, Kerrville, TX, United States of America; Yale University Yale School of Public Health, UNITED STATES

## Abstract

Leptospirosis, an emerging infectious disease caused by bacteria of the genus *Leptospira*, is thought to be the most widespread zoonotic disease in the world. A first step in preventing the spread of *Leptospira* is delineating the animal reservoirs that maintain and disperse the bacteria. Quantitative PCR (*q*PCR) methods targeting the *LipL32* gene were used to analyze kidney samples from 124 House mice (*Mus musculus*), 94 Black rats (*Rattus rattus*), 5 Norway rats (*R*. *norvegicus*), and 89 small Indian mongooses (*Herpestes auropunctatus*) from five cattle farms in Puerto Rico. Renal carriage of *Leptospira* was found in 38% of the sampled individuals, with 59% of the sampled mice, 34% of Black rats, 20% of Norway rats, and 13% of the mongooses. A heterogeneous distribution of prevalence was also found among sites, with the highest prevalence of *Leptospira*-positive samples at 52% and the lowest at 30%. Comparative sequence analysis of the *LipL32* gene from positive samples revealed the presence of two species of *Leptospira*, *L*. *borgpetersenii* and *L*. *interrogans* in mice, detected in similar percentages in samples from four farms, while samples from the fifth farm almost exclusively harbored *L*. *interrogans*. In rats, both *Leptospira* species were found, while mongooses only harbored *L*. *interrogans*. Numbers tested for both animals, however, were too small (n = 7 each) to relate prevalence of *Leptospira* species to location. Significant associations of *Leptospira* prevalence with anthropogenic landscape features were observed at farms in Naguabo and Sabana Grande, where infected individuals were closer to human dwellings, milking barns, and ponds than were uninfected individuals. These results show that rural areas of Puerto Rico are in need of management and longitudinal surveillance of *Leptospira* in order to prevent continued infection of focal susceptible species (i.e. humans and cattle).

## Author summary

Leptospirosis is a global zoonotic disease that recently has shown an increase of human cases in many regions, Puerto Rico being one of them. To decrease human *Leptospira* infections it is necessary to ascertain the role of animal reservoirs to maintain the pathogen in the environment. However, no studies of wild mammal reservoirs in Puerto Rico have been done since 1963. We addressed the prevalence of *Leptospira* in four species of introduced wild mammals, which included three commensal rodent species (*Mus* and *Rattus* spp.) and the small Indian mongoose (*Herpestes auropunctatus*) on dairy farms in Puerto Rico. Pathogen prevalence ranged from high to moderate in mice and mongooses, respectively. We also found that the pathogen is not distributed homogeneously among the sites we sampled and that landscape features, both natural and manmade, can play a role in the distribution of *Leptospira*. Our study provides an initial exploration of wild mammal reservoirs for *Leptospira* in Puerto Rico, and our data highlight the need for management of these species to potentially decrease pathogen transmission rates in both humans and cattle.

## Introduction

Along with increasing globalization, climate change, and urban expansion, the rising number of emerging infectious diseases is a major concern. Among emerging infectious diseases, over 60% are multi-host zoonoses many of which are classified as “neglected” owing to a general lack of knowledge about their epidemiology, more so in the tropics [1]. Perhaps the most widespread neglected zoonotic disease in the world is leptospirosis, which has an estimated annual global incidence of 1.03 million human cases with a projected number of 60,000 as fatal cases [2, 3]. Leptospirosis is caused by spirochetes of the genus *Leptospira* [4], with at least 15 known pathogenic species possessing over 250 serovars [5–7]. In known natural reservoirs, such as dogs, rodents, and cattle, *Leptospira* persists and multiplies within the renal tubules from which they are dispersed by urination of the moving hosts throughout the local landscape [4, 6]. Once in soil and water, these bacteria can remain viable for several months and can infect susceptible species through open-skin wounds and mucus membranes [5, 8]. Humans are incidentally infected with *Leptospira* following exposure to soils or water that is contaminated with animal urine [6].

Leptospirosis is an endemic disease in South Pacific island countries [9–11]. It is also widespread in the Caribbean islands including Haiti, Jamaica, Martinique, and Trinidad and Tobago [12–14]. However, incidence and prevalence of leptospirosis are largely underestimated throughout tropical environments especially since clinical signs associated to other febrile diseases, such as with dengue, malaria, and Zika, are strikingly similar; therefore, diagnosis and treatment is problematic [15–19]. In Puerto Rico, leptospirosis was first suspected in 1918 and confirmed in 1939 [20]. Current data for this island are limited, but reported incidence has increased over the past decade [16, 21]. Leptospirosis has been known to cause abortions, birth complications, and reduced milk production in cattle [22–24]. Due to the shared environment and level of contact with the animals, livestock workers are at risk of contracting leptospirosis areas where the pathogen is present [21, 25, 26]. The dairy industry in Puerto Rico comprises up to 25% of agriculture-related income and is historically the most important agricultural commodity on the island [27, 28]. Employing leptospirosis prevention regimens for cattle does not completely eliminate the threat of contraction if wildlife reservoirs are maintaining this pathogen in the farm environment. Therefore, assessing the risk associated with potential wildlife vectors in rural farm areas of Puerto Rico will inform future plans that aim to reduce transmission rates. The first step to this approach is identifying the wildlife species that are potentially acting as reservoirs on and around farms.

One of the most effective methods for managing zoonotic disease outbreaks is managing the wildlife reservoirs responsible for spreading the disease [21, 29, 30]. Invasive and pest (i.e. commensal rodents) species are of particular concern, because they readily adapt to human activity and urban settings, which places them in closer proximity to humans [29, 31]. This can be particularly true in farm settings where rodents and other pests can have direct access to animal feed and bedding areas. The objective of this study was to provide data on the prevalence of *Leptospira* in four invasive and pest species on rural farms of Puerto Rico; namely, House mice (*Mus musculus*), two rat species (*Rattus rattus* and *R*. *norvegicus*), and small Indian mongooses (*Herpestes auropunctatus*), which are now ubiquitous throughout the island and known reservoirs for *Leptospira* [1, 12]. We sampled at five rural locations in different parts of Puerto Rico, analyzed kidney tissue for renal carriage of *Leptospira*, and correlated *Leptospira* presence and absence data to individual distances from signature features within each location.

## Methods

### Sampling sites and sample collection

**Fig 1:**
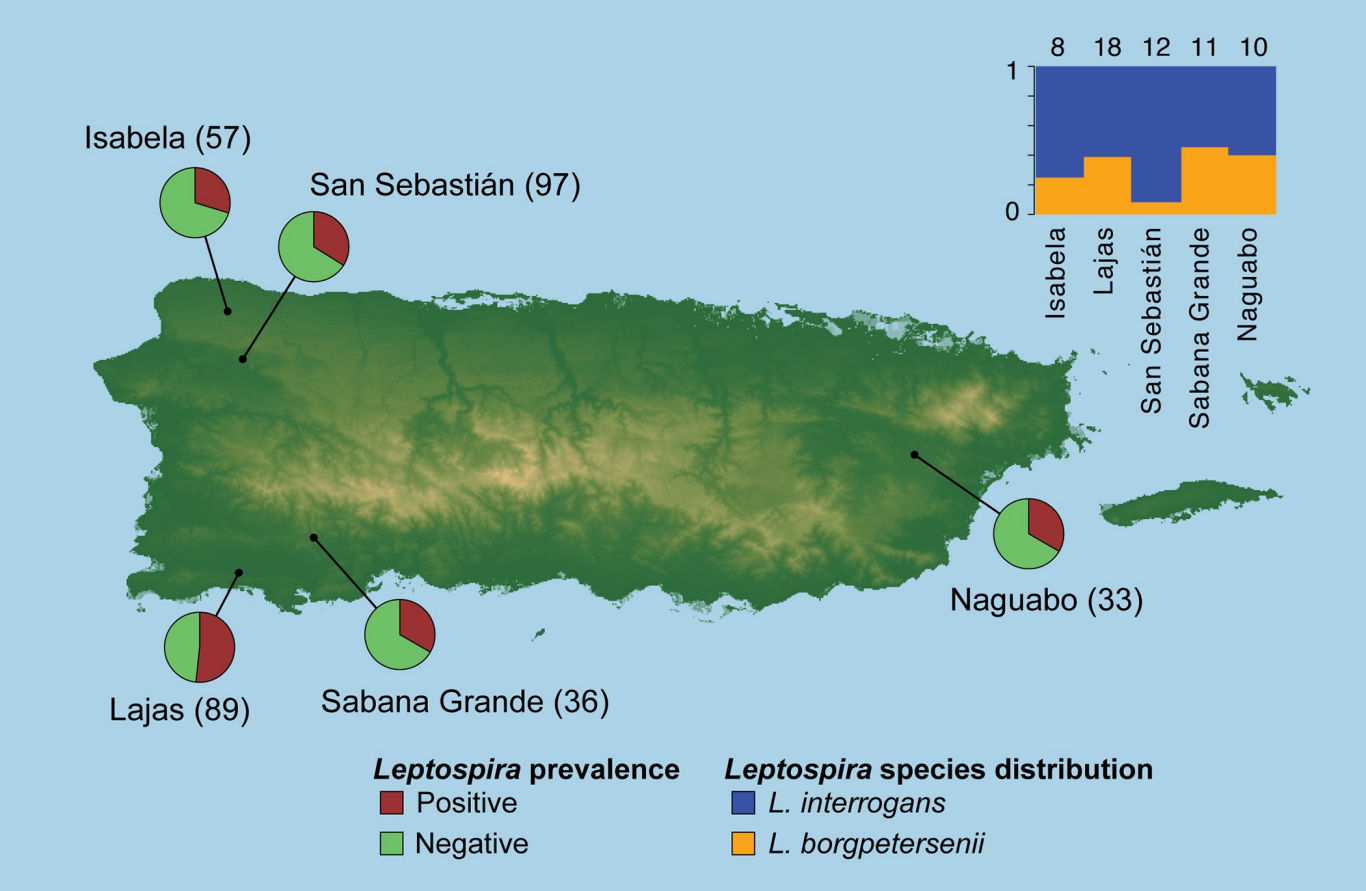
*Leptospira* prevalence for small invasive mammals (*Mus*, *Rattus*, and *Herpestes*) collected from five farms in Puerto Rico. Pie charts represent the total ratio of positive and negative individuals captured at each farm location. The bar chart (top right) represents the ratio of *L*. *interrogans* and *L*. *borgpertersenii* detected in the animal reservoirs at each site. Sample sizes for each farm location are indicated with parentheses. Prevalence per site is overlaid on an elevation map based on the Puerto Rico, PR 1 arc-second MHW DEM [68]. Open source raster accessed from: https://www.ngdc.noaa.gov/dem/squareCellGrid/download/1561.

Rodents and mongooses were trapped on cattle farms from five municipalities in Puerto Rico during the summers of 2014 and 2015 (Fig 1). Municipalities sampled for 2014 included dairy cow farms in Lajas (18.041189, -67.042908), Isabela (18.46116, -67.05652), San Sebastián (18.378265, -67.022423), and Naguabo (18.238525, -65.719208). During 2015, the same municipalities were sampled along with the addition of a beef cattle farm in Sabana Grande (18.036125, -66.931173). Lajas and Sabana Grande are both located in the southwestern Caribbean Sea side island and have a tropical savannah climate. Sampling sites San Sebastián and Isabela are on the northeastern Atlantic Ocean side of the island and they are characterized by a tropical rainforest climate. Naguabo represents the coastal east side of the island and receives the most amount of rain of the five farms. These sites formed part of a larger project focused on livestock health, with an emphasis on the impacts of Cattle Fever Ticks (CFTs, *Rhipicephalus* spp.) as pathogen vectors. Sampling for small mammals to ascertain their potential role as tick hosts opened the opportunity to collect samples for the present project. Selection of the farm sites was driven by the original goal of studying CFTs. Criteria for including farm in the study required the presence of the CFT as well as the willingness of farm owners to volunteer to participate in the study. An additional consideration was the distribution of these farms along different ecological zones from the island. Thus, it should be emphasized that farm selection was not driven by any previous information associated to presence of *Leptospira* in humans or cattle. More precisely the present project is ancillary to a larger one and thus of an exploratory nature.

Sherman live traps (3"x 3.5"x 9") (H.B. Sherman Traps, Inc., Tallahassee, FL, USA) baited with rolled oats, and Tomahawk Live Traps (20"x7"x7") (Tomahawk Live Traps, Hazelhurst, WI, USA) baited with tuna fish were placed in transects of either one line of 40 or two lines of 20 traps. Tomahawk traps were placed approximately 15–20 meters apart and Sherman traps were placed approximately 2–5 meters apart depending on the habitat being sampled, which included ecotones, grasslands, cattle pastures, riparian zones, and around human dwellings. The positions of captured animals were recorded with a GPS unit (Garmin Montana 650, Garmin Corp., Kansas City, KS, USA). Tomahawk traps were checked for captures throughout diurnal hours, three times a day (early morning, midday, and evening) to target mongooses. Tomahawk and Sherman traps were left overnight to target rodents.

Immediately after capture, animals were euthanized by cervical dislocation after first being rendered unconscious with isoflurane. Weight and size measurements of individuals were taken along with tissue samples that included kidneys, a liver fragment, the GI tract, heart, and lungs obtained using sterilized equipment. During summer 2014, tissue samples were stored in 70% ethanol (EtOH) and transferred to 95% EtOH at the end of the field season. During the summer of 2015, kidney samples were stored in 95% EtOH and kept cool at approximately 4°C throughout the field season. Samples were stored at different EtOH concentrations between years due to a lack of resources during 2014.

### Molecular analyses

DNA was extracted from kidneys using the Qiagen DNeasy Blood and Tissue Extraction Kit following the manufacturer’s instructions (Qiagen Inc., Valencia, CA, USA). As per these instructions, we extracted DNA from approximately 20mg of tissue, however this measurement was not systematically standardized. DNA extracts were stored frozen at -18°C. A *q*PCR TaqMan assay with primers designed to target the *LipL32* gene present in pathogenic *Leptospira* sp. was used to test for the presence of *Leptospira* [32, 33]. For the purpose of this exploratory study, *q*PCR was used for the detection of *Leptospira* but was not used for quantification. TaqMan based analyses were carried out on an Applied Biosystems StepOne Plus Real-Time PCR System (Applied Biosystems, Foster City, CA, USA) in duplicate in a volume of 25 μl containing 12.5 μl of TaqMan Fast Advanced Master Mix (Applied Biosystem, Foster City, CA, USA) 1 μl of each forward primer LipL32-45F (700 nM, ^5’^AAG CAT TAC CGC TTG TGG TG) and reverse primer LipL32-286R (700 nM, ^5’^GAA CTC CCA TTT CAG CGA TT), 1 μl of probe LipL32-189P (150 nM, ^5’^[6-FAM]- AA AGC CAG GAC AAG CGC CG -[BHQ1]), 1 μl of DNA template and 8.5 μl of water. An initial denaturation at 95°C for 5 minutes was followed by 40 cycles of 95°C for 15 seconds and 60°C for 30 seconds [32, 33]. *LipL32* gene amplicons obtained by end-point PCR with primers LipL32-45F and LipL32-286R from DNA of *Leptospira interrogans* serovar Copenhageni strain Fiocruz L1-130, kindly provided by Dr. Albert I. Ko (Yale University Schools of Public Health and Medicine, New Haven, CT), served as a positive control. Samples that amplified at ≤ 40 were considered positive. All samples were run in duplicate along with two negative controls. Assays were only considered valid if the negative control did not show an amplification signal.

Partial *LipL32* genes from a subset of positive samples were re-amplified by end-point PCR with primers LipL32-45F and LipL32-286R. Amplicons (242 bp) were sequenced on an Applied Biosystems Genetic Analyzer 3500xL (Life Technologies, Carlsbad, CA), and sequences deposited at Genbank. Sequences were assembled in Geneious 8.1.7 (Biomatters Ltd, Auckland, New Zealand), and checked in GenBank/EMBL databases using the BLAST algorithm [34]. This subset was selected to include representative groups of samples from all species and farm locations included in this study and represents approximately 50% of the identified positive samples.

### Landscape analyses

Landscape features included natural ponds, slurry ponds, milking areas within farms, and human buildings for which GPS positions were recorded. GPS positions of animals collected in the field were used to calculate the distance of each individual to landscape features. Average distances between these landscape features and positive or negative samples were then used in Welch’s t-test (unequal variances) to assess the relationships between prevalence of *Leptospira* in animals and landscape features on the farm. *Leptospira* presence/absence data were analyzed with SaTScan v9.4.2 software to test for significant clusters of positive cases on the landscape [35, 36]. Given that prevalence is binomial (infected or not) and presented as a proportion, we used Jeffrey’s confidence intervals for our estimates of prevalence among and within species and farms. We used the “prevalence” R package v.0.4.0 and an alpha value of 0.05 for significance assessments. A chi-squared test was conducted to determine if there was a heterogeneous distribution of *Leptospira* among the five farms.

### Ethics statement

Collection and handling of wild rodents followed the Guidelines of the American Society of Mammalogists for the use of wild mammals in research [37]. Our protocol was reviewed and approved by the Texas State University Institutional Animal Care and Use Committee (protocol #0514_0303_07) and scientific collecting permits for wild mammal collection were provided by Departamento de Recursos Naturales y Ambientales from Puerto Rico (2014-IC-063 to Ivan Castro-Arellano). Access to private property was granted by landowners.

## Results

### Prevalence of *Leptospira*

Over two trapping seasons, we sampled 312 mammals comprising of 124 house mice (*Mus musculus*), 94 black rats (*Rattus rattus*), 5 Norway rats (*R*. *norvegicus*), and 89 Small Indian mongooses (*Herpestes auropunctatus*), sampling and prevalence data are summarized in Table 1. Sample sizes per site ranged from 33 (Naguabo) to 97 (San Sebastián) with an overall *Leptospira* prevalence of 0.38 (0.33–0.44) across all species. Although sample sizes among species were not uniform the overall prevalence values among and within sites it is of epidemiological significance to understand the level at which *Leptospira* is present among all animal reservoirs for both the island and each farm. Lajas had significantly higher prevalence compared to the other sites (*x*^*2*^ = 9.97, df = 4, *p* < 0.04). San Sebastián, Naguabo, Sabana Grande, and Isabela, had a similar prevalence values (0.30–0.34) regardless of varying sample sizes (n = 33–97). Mice generally showed higher prevalence of *Leptospira* (0.42–0.70), followed by Black rats (0.00 to 0.61), and mongooses (0.00–0.21). Norway rats were caught at two sites and in low numbers (n = 5), only one individual was positive for *Leptospira*. Overall, detections of *Leptospira* in mice were significantly higher than in rats and mongooses (*x*^*2*^ = 42.347, df = 2, p < 0.0001).

**Table 1:** *Leptospira* prevalence, with Jefferys confidence intervals, among invasive small mammals sampled from cattle farms in Puerto Rico during summer 2014 and 2015. Positives columns represent numbers of infected individuals (outside parenthesis) and total sample size (*n*, in parenthesis). Jeff. C.I. = Jefferys Confidence Interval.

	Overall		*Mus musculus*		*Rattus rattus*		*Rattus norvegicus*		*Herpestes auropunctatus*	
Locality	positives (*n*)	Prevalence (Jeff. C.I.)	positives (*n*)	Prevalence (Jeff. C.I.)	positives (*n*)	Prevalence (Jeff. C.I.)	positives (*n*)	Prevalence (Jeff. C.I.)	Positives (*n*)	Prevalence (Jeff. C.I.)
Lajas	46 (89)	0.52 (0.41–0.62)	26 (37)	0.70 (0.54–0.83)	14 (23)	0.61 (0.41–0.79)	–	–	6 (29)	0.21 (0.09–0.38)
San Sebastián	33 (97)	0.34 (0.25–0.44)	20 (34)	0.59 (0.42–0.74)	10 (39)	0.26 (0.14–0.41)	0 (3)	0.00 (0.00–0.44)	3 (21)	0.14 (0.04–0.33)
Naguabo	11 (33)	0.33 (0.19–0.50)	10 (18)	0.56 (0.33–0.76)	0 (7)	0.00 (0.00–0.23)	–	–	1 (8)	0.12 (0.01–0.45)
Sabana Grande	12 (36)	0.33 (0.20–0.50)	10 (24)	0.42 (0.24–0.61)	2 (5)	0.40 (0.09–0.79)	–	–	0 (7)	0.00 (0.00–0.23)
Isabela	17 (57)	0.30 (0.19–0.42)	7 (11)	0.64 (0.35–0.86)	7 (20)	0.36 (0.17–0.57)	1 (2)	0.50 (0.06–0.94)	2 (24)	0.08 (0.02–0.24)
**Overall**	**119 (312)**	**0.38 (0.21–0.49)**	**73 (124)**	**0.59 (0.50–0.67)**	**33 (94)**	**0.34 (0.25–0.45)**	**1 (5)**	**0.20 (0.02–0.63)**	**12 (89)**	**0.13 (0.08–0.22)**

### *Leptospira* diversity

Endpoint PCRs of *Leptospira* positive samples for *LipL32* gene fragments (242 bp) from 45 mice, 7 rats, and 7 mongooses generated a sufficient number of amplicons to allow automated Sanger sequencing (MK328816–MK328874). Sequences showed either 100% identity to GenBank reference sequences for *L*. *borgpetersenii* (KF928037) or *L*. *interrogans* (U89708, DQ149595). Overall *L*. *interrogans* (68%) was detected more among small mammals compared to *L*. *borgpetersenii* (32%) (t = -2.58, df = 7.9, p = 0.03). Both *Leptospira* species were detected in similar proportions in Lajas, Naguabo, Sabana Grande, and Isabela, while samples from the fifth farm San Sebastián harbored mainly *L*. *borgpetersenii* (Fig 1, Table 2). In rats, both *Leptospira* species were found, while mongooses only harbored *L*. *interrogans* (Table 2).

**Table 2:** *Leptospira* species infecting invasive small mammals captured on Puerto Rican cattle farms during summer 2014 and 2015. Assignments were inferred using *LipL32* sequence data from renal tissue.

Locality	n	*L*. *borgpetersenii*^a^	*L*. *interrogans*^*a*^
Lajas	18	7 (5, 2, 0)	11 (6, 1, 4)
San Sebastián	12	1 (0, 1, 0)	11 (10, 0, 1)
Naguabo	10	4 (4, 0, 0)	6 (5, 0, 1)
Sabana Grande	11	5 (5, 0, 0)	6 (5, 1, 0)
Isabela	8	2 (2, 0, 0)	6 (3, 2, 1)
**Overall**	**59**	**19 (16, 3, 0)**	**40 (29, 4, 7)**

^a^ Total (*Mus*, *Rattus*, *Herpestes*)

### Association of *Leptospira* with landscape features

Association of infection with landscape features was significant in Naguabo, where infected individuals tended to be closer to chosen landscape features **(**including a human dwelling, dairy cow milking area, and a pond) than were uninfected individuals (Table 3). At Sabana Grande, infected individuals were closer to the human dwelling than were uninfected individuals (Table 3). At the other locations, i.e. Lajas, San Sebastián and Isabela, infected and uninfected individuals did not differ in distance to the anthropogenic landscape features.

**Table 3:** Correlation of landscape features present on individual farms in Puerto Rico to *Leptospira* prevalence among host species collected from five farms in Puerto Rico during summers 2014 and 2015. Host species included *Rattus rattus*, *R*. *norvegicus*, *Mus musculus*, and *Herpestes auropunctatus*. Distances between host species and selected landscape features were calculated from GPS coordinates using the haversine equation. Welch’s t-tests were used to determine if the average distance of positive samples to the landscape features were closer than the average distance of negative samples using an aloha value of 0.05. Significant results are denoted in bold.

**Location**	**Feature**	**Distance (m) to negatives**^1^	**Distance (m) to positives**^2^	**Df**	**T**	**P**
Lajas						
	Human dwelling	2043.12	2237.99	80.95	0.70	0.48
	Human dwelling	1339.47	1038.90	80.81	-0.97	0.34
	Milking area	1390.05	1076.59	80.84	-0.97	0.34
	Pond	1646.12	1466.84	79.87	-0.68	0.50
	Wetland	1452.45	1174.42	80.48	-0.85	0.39
San Sebastián						
	Milking area	392.33	346.09	61.70	-0.85	0.40
	Wetland Pond	454.29	537.13	69.53	1.10	0.27
	Slurry Pond	414.85	380.96	60.44	-0.67	0.50
Naguabo						
	**Human dwelling**	**278.93**	**126.35**	**23.94**	**2.60**	**<0.02**
	**Milking area**	**234.42**	**100.71**	**24.00**	**2.53**	**<0.02**
	**Slurry Pond**	**254.38**	**128.19**	**23.95**	**2.44**	**<0.05**
Sabana Grande						
	**Human dwelling**	**337.88**	**133.45**	**28.98**	**-2.28**	**<0.05**
	Stock Pond	450.78	314.50	28.90	-1.37	0.18
Isabela						
	Horse Stables	565.04	571.06	29.23	-0.05	0.96
	Milking area	520.26	534.54	29.45	-0.13	0.90
	Slurry Pond	524.93	559.57	29.94	-0.33	0.74
	Stock Pond	601.45	620.19	29.23	-0.13	0.89

^1^Average distance of features to captured animals testing negative for *Leptospira*

^2^Average distance of features to captured animals testing positive for *Leptospira*

**Fig 2:**
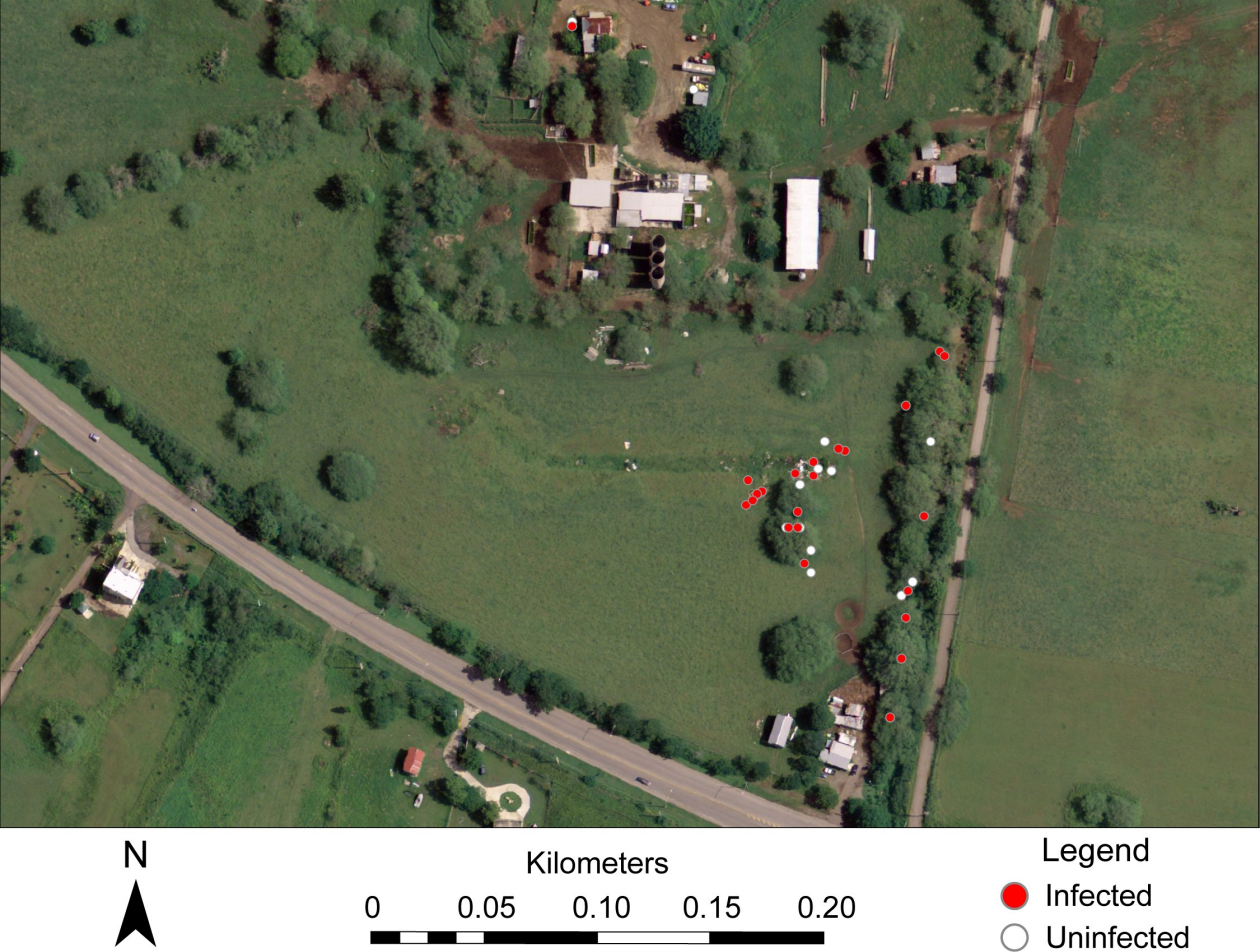
Potential cluster of reservoir animals (*Herpestes auropunctatus*, *Rattus* spp., *Mus musculus*) that tested positive for *Leptospira* at the farm location in the municipality of Lajas. Each point indicates the location of one individual. Map image includes the main milking and processing area for the farm. Structures in view include the milking area and a human dwelling. Base map for creating this figure was taken from an open source (https://datagateway.nrcs.usda.gov/GDGOrder.aspx).

Spatial analyses with SaTScan using a Bernoulli model identified four clusters within the studied farms but none were statistically significant. However, a cluster identified at the Lajas farm, although not significant at the level (p < 0.05) we had chosen, had a substantially different p-value than the other clusters (Fig 2 and Table 4). This cluster was in a field in close proximity to the milking area (0.14 km SE) and a building (0.15 km SW).

**Table 4:** Spatial clustering of samples positive for *Leptospira* at four locations, analyzed in SaTScan using a Bernoulli model. Analyses included *Herpestes auropunctatus*, *Rattus* spp. and *Mus musculus* collected from cattle farms in Puerto Rico. All positive samples throughout sampling sites were analyzed to determine if more positive samples that expected were detected in any given area using ci-square analyses.

	**Site**	**Radius**	**Expected**	**Observed**	**p**
**Cluster 1**	Lajas	0.029	5.49	12	0.07
**Cluster 2**	Sabana Grande	0.024	1.57	4	0.95
**Cluster 3**	Naguabo	0.086	1.57	4	0.95
**Cluster 4**	San Sebastián	0.029	2.75	6	0.97

## Discussion

Although all four species of mammals analyzed in our study tested positive for *Leptospira*, prevalence of *Leptospira* was much higher in mice than in mongooses or both Black or Norway rats (Tables 1 and 2). Results from this study suggest that mice potentially play a more important role as reservoir for *Leptospira* in rural parts of Puerto Rico than rats. However, since mice excrete less urine than rats they are likely also shedding fewer *Leptospira* into the environment. A major limitation to this study was not quantifying *Leptospira* load among individuals. Since this was an exploratory study that aimed to identify a potential health risk to cattle and farmworkers on Puerto Rican dairy farms, the main goal was to verify the presence of the pathogenic *Leptospira* spp. among potential reservoirs present at each farm. Now that the presence has been verified, future studies on farms should concentrate on accurately quantifying the load of reservoir animals. Quantifying pathogen load will provide a better idea of the extent to which each species contributes to *Leptospira* maintenance in the environment.

In Puerto Rico, all four animal species we sampled had been identified as reservoirs for *Leptospira* before, with high prevalence, i.e. 48% in House mice, 37% in Black rats, 40% in Norway rats, and 20% in small Indian mongooses [20]. In a previous study, both rat species were found to carry *Leptospira*, at a prevalence of 39% [38]. Although both of these studies were limited to the urban area of San Juan, our data confirm that House mice, black rats, and Norway rats are also important reservoirs in rural areas of Puerto Rico with high *Leptospira* prevalence in all animal species.

It should be noted that few Norway rats were caught in sampled farms. Previous studies have found large numbers and with high prevalence of *Leptospira* in urban areas [39, 40]. In both tropical and temperate urban areas, Norway rats are frequently encountered and infected with *Leptospira*, with prevalence values often around 40% [41–43], but up to 89% as well [44, 45]. In our study, Norway rats were rarely encountered and caught (n = 5), which was likely a consequence of this species preferring urban areas over rural areas [46]. Thus, our prevalence values of 20% that reflected detection of *Leptospira* in one individual only, are without statistical significance though still similar to other published data [47, 48].

High prevalence of *Leptospira* has been shown for different species of mongooses in previous studies [49–51]. Our study resulted in few detections of *Leptospira* in Indian mongooses at any site on Puerto Rico corresponding to an overall prevalence rate of 13%. Results on other Caribbean islands like Barbados, however, show much higher prevalence, with prevalence values close to 41% in mongooses, while mice were infected at much lower prevalence (28%) [51]. Even though similar numbers of mongooses and mice were tested in this study, the results do not match our data with 13% mongooses harboring *Leptospira*, and mice being the most infected with 59% prevalence. The result trends, however, are similar to numbers ascertained from a previous survey conducted in San Juan, Puerto Rico [20].

Two species of *Leptospira*, i.e. *L*. *interrogans* and *L*. *borgpetersenii* were detected in mice and rats, while mongooses only harbored *L*. *interrogans*. The lack of detection of *L*. *borgpetersenii* in mongooses, however, is potentially a function of the small sampling size used for species analyses of *Leptospira*, i.e. seven individuals from 4 locations. Since a higher prevalence of *L*. *borgpetersenii* is seen in both rats and mice, it could also be that mongooses are not regularly coming into direct contact with this pathogen due to mongooses living in lower densities than mice or rats and the short environmental persistence of *L*. *borgpetersenii* outside of the host. *L*. *borgpetersenii* is thought to survive poorly in the environment due to point mutations in environmental sensing and metabolite transport and utilization genes, and thus is transmitted most frequently through direct contact [52]. In contrast, *L*. *interrogans* survives for extended times in the environments [53] and is transmitted readily through contact with surface waters [54, 55]. *L*. *borgpetersenii* (i.e. serovar Hardjo) has been reported as most prominent *Leptospira* sp. in cattle in Chile [56], and cattle were proposed as maintenance host [24]. *L*. *borgpetersenii* has been detected in cattle from other countries, however, equally often in other animals, including many different rodent species [57]. Thus, while the tropical conditions on Puerto Rico generally favor environmental survival and transmission of *Leptospira* [53], animal host preferences of different *Leptospira* species could not be established in our study.

Both *Leptospira* species identified in this study are known to persist in urban rat populations, as demonstrated for samples from Malaysia [58]. Both species were detected in mice, in similar percentages in four of the five sampling locations in Puerto Rico, while samples from the fifth location, San Sebastián, almost exclusively harbored *L*. *interrogans*. Since there were no apparent landscape features ecologically isolating this location from the other study locations, it is interesting that there was an absence of *L*. *borgpetersenii* in mice. Low abundance and small sampling size affecting detection of *L*. *borgpetersenii* is not a likely explanation, since sampling size was similar to those of the other locations and *L*. *borgpetersenii* was identified in the only rat sample analyzed from this location.

In some farms, such as those is Barbados, agricultural workers were identified as having a high risk of contracting leptospirosis due to their proximity to contaminated water and soil [59], and in an urban slum in Brazil lower elevations were related to higher *Leptospira* concentrations [55]. Furthermore, previous studies have associated the persistence of Leptospira with moist environments [55, 60]. Naguabo was the only location in this study for which several landscape feature-*Leptospira* prevalence relationships were found. These relationships are, in part, possibly due to the location of the farm, i.e. a valley in a mountainous area in close proximity to the El Yunque National Rainforest which experiences an average rainfall of approx. 2,134 mm which is much higher than in all of the other sampling sites. Heavy rainfall results in runoff that might carry bacteria to and concentrate them at structures located in areas with the lowest elevation of this farm and in a nearby stream. Here, the moist environments would provide suitable conditions for some species of *Leptospira* to persist for longer lengths of time; therefore, rodents would have a greater opportunity to come into contact with the pathogens. Ponds, milking areas, and human dwellings are also desirable rodent habitats due to providing easier access to resources, so rodents are more likely to persist in higher abundances in close proximity to these areas.

The relationship between landscape features and the distance of positive and negative samples were inconsistent between the five farms included in this study. While it is generally thought that *Leptospira* is associated with the presence of environmental features such water bodies, data presented in this study do not support this hypothesis. This inconsistency could be due to interspecific interactions among animal reservoirs having a greater effect on *Leptospira* prevalence than the environmental features themselves. Ansersen-Ranberg et al. (2016) found that although some groups of animal reservoirs had similar prevalence values, these were inconsistently correlated to environmental factors [61]. Additionally, there is some indication that sociality in reservoir species or human effects on landscape has the potential to create hot spots for *Leptospira* presence. While not statistically significant, the spatial cluster of positive samples detected in the Lajas farm (Fig 2) held some ecological and epidemiological significance because it coincided spatially with an area where discarded farm materials (cut tree branches, tires, metal pieces, etc.) had been deposited as a pile in the middle of an open field. This created a habitat suitable for mice where individuals, both positive and negative, congregated (Fig 2) likely raising pathogen transmission among those individuals. As a precaution, farmers were informed that waste management around farm buildings to prevent rodent infestations should be a high priority to avoid concentration of *Leptospira* positive individuals. Since it is likely that the small sample size and uneven coverage of the farm influenced the significance of the cluster analysis, future research should aim to increase samples size and sample coverage at the individual farms to better identify potential disease hotspots.

**Fig 3:**
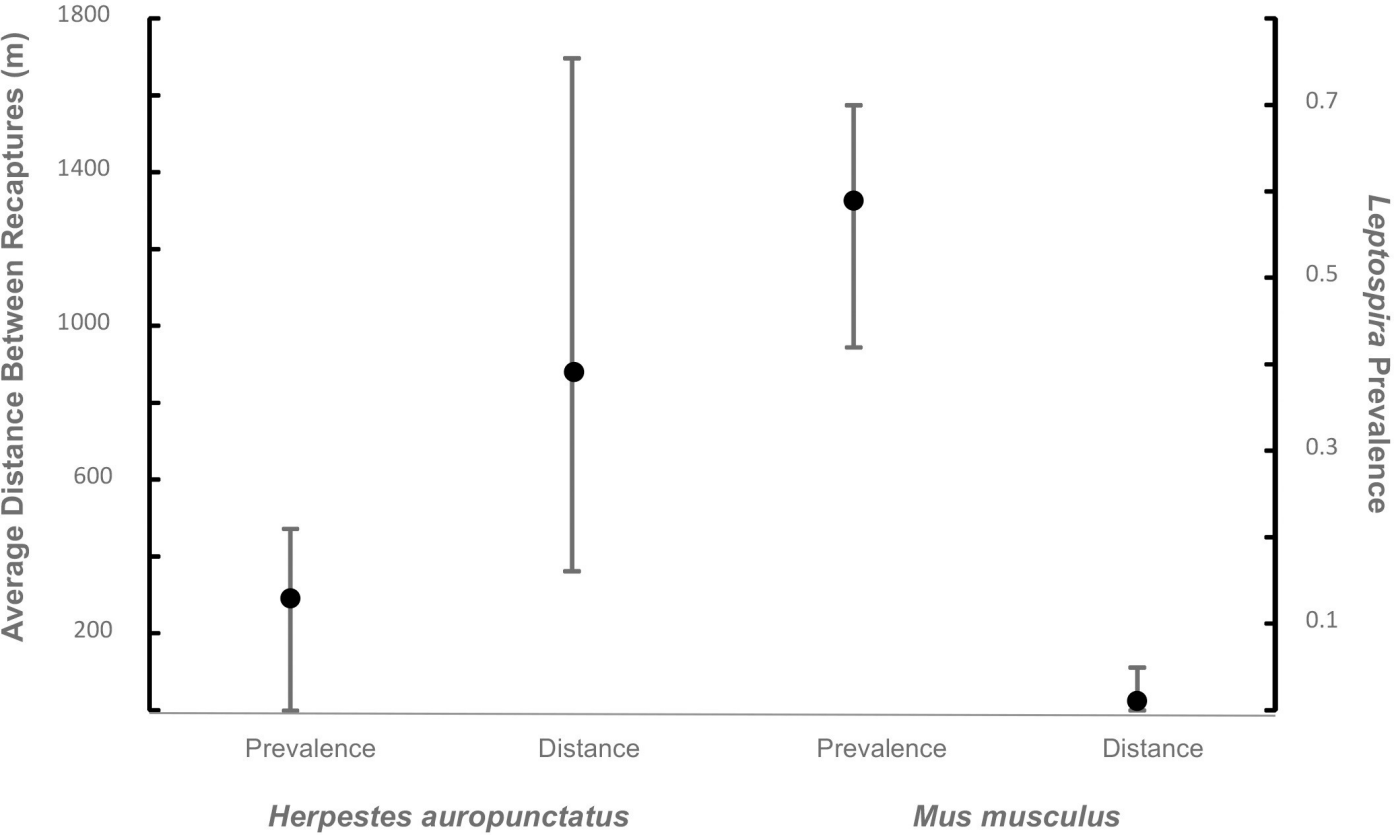
Comparison of *Leptospira* prevalence and movement distances of *Herpestes auropunctatus* and *Mus musculus*. Spatial movement data of *H*. *auropunctatus* was collected via a parallel telemetry sampling on four of the dairy farms in Puerto Rico that formed part of present study. Detailed results from this telemetry study forms part of a larger project about zoonotic diseases in cattle farms in the island and will be published elsewhere. The average distance between telemetry fixes among mongoose individuals equaled 362.61 meters, ranging from 362.61 meters– 1696.5 meters (extreme values represented with bars around point that represents average). Spatial movements of *M*. *musculus* were retrieved from a published review [69] that included capture-recapture studies for *M*. *musculus* in predominantly rural settings and included both feral and commensal house mice. The average distance between recaptures in this review equaled 25.27 meters, and the range was 0.9 meters to 112 meters (extreme values represented with bars around point that represents average). Prevalence estimates for both species represented in this graph are from the current study (extreme values represented by bars with averages as points).

Another issue that needs to be addressed is the dynamics of interspecific transmission and pathogen maintenance at each farm and among farms in a landscape. The contrast between usual spatial movements and *Leptospira* prevalence between mongooses and mice shows a clear opposite pattern (Fig 3) in which mice usually travel short distances but show greater pathogen prevalence whereas the opposite is true for mongooses. Our study found a higher prevalence of *Leptospira* in mice thus pointing out a potential important role for this species to maintain the pathogen at a given site. However, this rodent species is usually found in close association to human dwellings and likely will not be a relevant factor to spread the pathogen to other sites. In contrast, mongooses readily travel longer distances and would be capable of spreading *Leptospira* among adjoining farms and further into areas of high human use, a behavior reported for other Herpestidae species that are also reservoirs for this pathogen [50]. Since small Indian mongooses readily prey on commensal rodents a high opportunity of contact between these species exist so the need to evaluate this as a potential route for interspecies transmission and its role in maintaining the pathogen in Puerto Rico deserves further evaluation. Future studies that investigate this possible relationship should also consider other factors, such as host carriage rate, host urine extraction rates, and pathogen life history traits to gain a clear picture of how much host behaviors potentially affect prevalence between farms.

According to news sources, after hurricane Maria landed in September of 2017 this event increased the number of leptospirosis infections in humans across Puerto Rico. This was likely because people were obligated to drink contaminated water as a result of failed infrastructure. As many as 76 individuals were likely infected and a small handful of these were fatal cases [62]. As climatic events such as this increase in intensity and frequency as a result of climate change, it is becoming increasingly important to monitor the epidemiological consequences on pathogenic agents of neglected infectious diseases. This is especially true in tropical areas such as Puerto Rico because these are the most severely affected by intense climatic events such as hurricanes and monsoons, as was illustrated with Hurricane Maria. This study demonstrates the need for leptospirosis monitoring programs to be implemented in rural areas of Puerto Rico along with urban areas.

In conclusion, this study established baseline data on the prevalence of *Leptospira* species in four animal species in five rural areas, at both the east and west coasts of Puerto Rico. The capture of large numbers of rodents and mongooses with high prevalence of *Leptospira* in animals from all locations supports suggestions for the implementation of management plans for rodent and mongoose control to reduce the risk of susceptible focal species (i.e. humans and cattle) to contract *Leptospira*. These management plans could focus on all or selected animal species depending on their abundance at the respective location, and should include monitoring prevalence of *Leptospira* in cattle and adjacent soils and waters to assess environmental risks of infection in rural areas of Puerto Rico [54, 55, 63–67].
